# Innate Response Activator (IRA) B Cells Reside in Human Tonsils and Internalize Bacteria *In Vitro*

**DOI:** 10.1371/journal.pone.0129879

**Published:** 2015-06-12

**Authors:** Nico Chiappini, Rocco Cantisani, Laura Pancotto, Paolo Ruggiero, Domenico Rosa, Andrea Manetti, Antonio Romano, Francesca Montagnani, Sylvie Bertholet, Flora Castellino, Giuseppe Del Giudice

**Affiliations:** 1 Novartis Vaccines and Diagnostics (a GSK company), Research Center, Via Fiorentina 1, Siena, Italy; 2 Otorhinolaryngology Department, University Hospital Le Scotte, Strada delle Scotte 14, Siena, Italy; 3 Department of Medical Biotechnologies, Infectious Unit, University Hospital Le Scotte, Strada delle Scotte 14, Siena, Italy; COCHIN INSTITUTE, Institut National de la Santé et de la Recherche Médicale, FRANCE

## Abstract

Innate response activator (IRA) B cells have been described in mice as a subset of B-1a B cells that produce granulocyte/macrophage colony-stimulating factor (GM-CSF) and have been found in the spleen upon activation. In humans, identification, tissue localization and functionality of these lymphocytes are poorly understood. We hypothesized that IRA B cells could reside in human palatine tonsils, which are a first line of defense from infection of the upper respiratory tract. In the present work, we used flow cytometry and confocal microscopy to identify and characterize human IRA (hIRA) B cells in tonsils. We show that CD19^+^CD20^+^GM-CSF^+^ B cells are present in the tonsils of all the subjects studied at a frequency ranging between ~0.2% and ~0.4% of the conventional CD19^+^CD20^+^GM-CSF^-^ B cells. These cells reside within the B cell follicles, are mostly IgM^+^IgD^+^, express CD5 and show phagocytic activity. Our results support a role for hIRA B cells in the effector immune response to infections in tonsils.

## Introduction

B lymphocytes are key players in adaptive immune response due to their ability to differentiate into cells producing antigen-specific antibodies following encounter with micro-organisms or vaccination. B cells have been classified into various sub-populations including memory, germinal center and follicular B cells, each identified by particular phenotypic arrays of surface markers. Together, these populations constitute conventional B cells (or B-2 B cells) which react adaptively to antigen challenges with antibody responses after differentiation in plasma cells by affinity maturation [1]. In recent years, other populations of B cells have been described and classified as components of the innate immune system [2]: marginal zone (MZ) B cells, specialized in responses to blood-borne pathogens; B-1 B cells, which constitutively and spontaneously secrete “natural” antibodies necessary as first line of defense against infections [3], and B-10 B cells, with immunosuppressive function mediated by the production of IL-10 [4].

A new subpopulation of B lymphocytes, called Innate Response Activator (IRA) B cells, has been described in mice. They can be identified by the expression of CD19^+^IgM^+^CD5^+^CD43^+^ and the ability to produce granulocyte–macrophage colony-stimulating factor (GM-CSF). These murine cells represent a transitional B-1a-derived population, reside in peritoneal and pleural cavities during the steady state, respond quickly after infection, and expand in the spleen during sepsis (or LPS stimulation) [5,6] and atherosclerosis [7], and in lung fluid in a lung infection model [8].

The production of GM-CSF by IRA B cells may exert different effects, depending on the pathology and on the compartments where they reside. During the onset of intestinal sepsis, IRA B cells may participate in neutrophil-dependent bacterial clearance [5], while in atherosclerosis they may promote the expansion of classical dendritic cells (DCs) [7]. Furthermore, GM-CSF signaling may have an autocrine effect on IRA B cells intervening in the auto-regulation of IgM production [8]. However, most of the work on IRA B cells has been conducted in the spleens and peritoneal/pleural cavities of mice; limited information is yet available in humans.

We were interested in (i) evaluating whether IRA B cells could be identified in human palatine tonsils that, as strategic secondary lymphoid organs, represent a first line of defense against invasive microorganisms in the upper respiratory tract; (ii) characterizing them phenotypically, and (iii) investigating their potential function.

## Materials and Methods

### Human subjects

We recruited patients undergoing tonsillectomy at the Otorhinolaryngology Unit of the University Hospital of Siena (Siena, Italy). Eligible tonsillectomized patients were clinically stable children (aged ≤ 16 years) with recurrent tonsillitis. Enrolment criteria were: ≥ 7 well-documented, clinically important, adequately treated episodes of throat infection in the preceding year, or ≥ 5 such episodes in each of the two preceding years, or ≥ 3 such episodes in each of the three preceding years. Written informed consent for each single patient was obtained from the next of kin, caretakers, or guardians of the minors involved in the study, which was approved by the ethical committee of Siena University.

### Preparation of single-cell suspensions from tonsils

Tonsil tissues were maintained in cold HBSS medium in a plastic container until processing (within 1 to 3 h after surgery). Using a sterile scalpel, the tissue was fragmented in a Petri dish with 2 ml HBSS + 700 U/ml collagenase (Gibco) + 20 μg/ml DNase (Sigma). Samples were incubated for 40 min at 37°C. After enzymatic digestion, tonsil-derived cell suspensions were filtered twice through 70-μm diameter cell-strainers, washed, and suspended in 20 ml HBSS. Mononuclear cells were stratified on Ficoll-Paque PREMIUM (GE Healthcare), and centrifuged for 30 min at 1000×g at room temperature. The interphase ring containing mononuclear cells was transferred in 10 ml of HBSS; after 2 washes, mononuclear cells were counted with a Vi-cell XR counter (Beckman Coulter).

### Flow Cytometry

The following anti-human antibodies were used for flow cytometry analysis: anti-CD19-BV605 SJ25C1, anti-CD20-APC-H7 2H7, anti-CD5-PECy5 UCHT2, anti-CD11b-V450 ICRF44, anti-CD27 PECy7 M-T271, anti-CD43-FITC 1G10, anti-CD183-PECy5 1C6, anti-CD185(CXCR5)-A488 RF8B2, anti-CD184-PECy5 12G5 (BD Pharmingen); anti-IgM-PE-CF594 G20-127, anti-IgG-V450 G18-145 (BD Horizon); anti-CD20-PerCP-Cy5.5 L27 (BD Biosciences); anti-CD93-PE R3, anti-IgD-PerCP-eFluor710 IA6-2, anti-CD11c-APC-eFluor780 BU15, anti-GM-CSF-eFluor660 GM2F3, anti-CD40-eFluor450 5C3 (eBioscience); anti-IgA-FITC IS11-8E10 (Miltenyi Biotec)anti-CD86-FITC 2331 (FUN-1) (BD Pharmingen); anti-CD284-AlexaFluor488 HTA125 (eBioscience). The following anti-human isotype controls were used: anti-IgG1-FITC, anti-IgG1-PECy5, anti-IgG1-PECy7 MOPC-21, anti-IgG2b-A488 A95-1, anti-IgG2a-PECy5 G155-178 (BD Pharmingen); anti-IgG1-V450 MOPC-21, anti-IgG1-PE-CF594 X40 (BD Horizon); anti-IgG1-eFluor450, anti-IgG1-eF660, anti-IgG1-APC-eFluor780 P3.6.2.8.1, anti-IgG1-PE P3, anti-IgG2a-PerCP-eFluor710 eBM2a, (eBioscience); anti-IgG1-FITC IS5-21F5 (Miltenyi Biotec); anti-IgG1-AlexaFluor488 (eBioscience). Acqua Live/Dead Stain (Life Tecnhnologies) was used to select live cells. For intra-cytoplasmic staining, cells were permeabilized and fixed using the Cytofix/Cytoperm Plus kit (BD Biosciences). Data were acquired on either LSRII special order or Fortessa flow cytometers (BD Biosciences), and analyzed with FlowJo (v8.8.6/v9.7.2; Tree Star).

### *In vitro* cultures

Tonsil-derived cells (5x10^5^/200 μl) were plated in medium alone (RPMI supplemented with 10% fetal calf serum, 2 mM l-glutamine, 10 mM Hepes, 100 U/ml penicillin, 100 μg/ml streptomycin, 1 mM sodium pyruvate, and 1× nonessential amino acids), or in medium plus 10 μg/ml CpG (Primm), 10 μg/ml goat F(ab')2 anti-human IgM-UNLB (SouthernBiotech), or *Staphylococcus aureus (S*. *aureus)* Cowan I (SAC, Calbiochem) at the final dilution of 1:20000. The incubation was performed in the presence of 1000 U/ml IL-2 (Peprotech) for 2 days at 37°C with 5% CO_2_. Brefeldin-A (Sigma) was added at 5 μg/ml 3 h before fixation and staining. Alternatively, 1.5 x 10^6^ cells/200μl RPMI supplemented with 10% fetal calf serum were plated in a 96-well plate and immediately stained using the antibodies listed above.

### Uptake of bacteria *in vitro*

5 x 10^5^ (300 μl/well) tonsil-derived cells were seeded on a poly-L-lysine-coated coverslip in a 24-well plate. Cells were incubated for 2 h at 37°C or 4°C with or without heat-inactivated *S*. *aureus* or PhRodo Green *S*. *aureus* Bioparticles at a MOI of 10:1 (bacteria:cells). After incubation, cells were fixed with 2% formaldehyde for 30 min at RT, washed with PBS and permeabilized with 0.1% Triton X-100. Coverslips were then washed again with PBS and blocked with 3% BSA—10% goat serum (Invitrogen) for 20 min at RT. Finally, coverslips were washed with PBS and incubated for 30 min at RT with antibody mix containing human anti-CD19-V450 HIB19 (BD Pharmingen), anti-GM-CSF-eFluor660 GM2F3, anti-MPO-PE 455-8E6 (eBioscience), all diluted in 0.1% BSA + 1% goat serum. After incubation, coverslips were washed and mounted on a slide with Gold Anti-Fade reagent (Life Technologies) for analysis by confocal microscopy.

### Immunofluorescence on tonsil cryosections

A portion of each tonsil was collected in dry conditions, immediately frozen in liquid nitrogen, and stored at -80°C until processing. Tonsil cryosections (8-μm thick) were obtained with the cryo-microtome CM1950 (Leica), fixed with 4% formaldehyde for 10 min at RT and washed three times with PBS. Sections were blocked with 1 mg/ml of human immunoglobulins (Gentaur) for 30 min at RT, washed with PBS and stained with an antibody mix containing anti-human CD19-V450 HIB19 (BD Pharmingen), anti-CD3-PE SK7 (BD bioscience), anti-GM-CSF-eFluor660 GM2F3 (eBioscience), anti-IgD-BrillantViolet421 IA6-2 (Biolegend), all diluted in 1 mg/ml human immunoglobulins for 1 h at RT. After incubation, sections were washed again with PBS and mounted with a coverslip using Gold anti-Fade reagent (Life Technologies) for analysis by confocal microscopy.

### Confocal microscopy

Images from human tonsil samples were acquired by Axio Observer LSM700 confocal microscope (Zeiss) at 20°C, using Plan-Apochromat 40X or 100X objective lenses with 1.3 numerical aperture. The 40X and 100X objective lens was used with the Zeiss Immersol 518F imaging medium. Images were processed with Zen 2008 software (Zeiss).

## Statistical analysis

Statistical differences between groups were evaluated using the non-parametric Mann–Whitney test. P-values ≤ 0.05 were considered statistically significant.

## Results and Discussion

### IRA B cells are present in human tonsils

**Fig 1:**
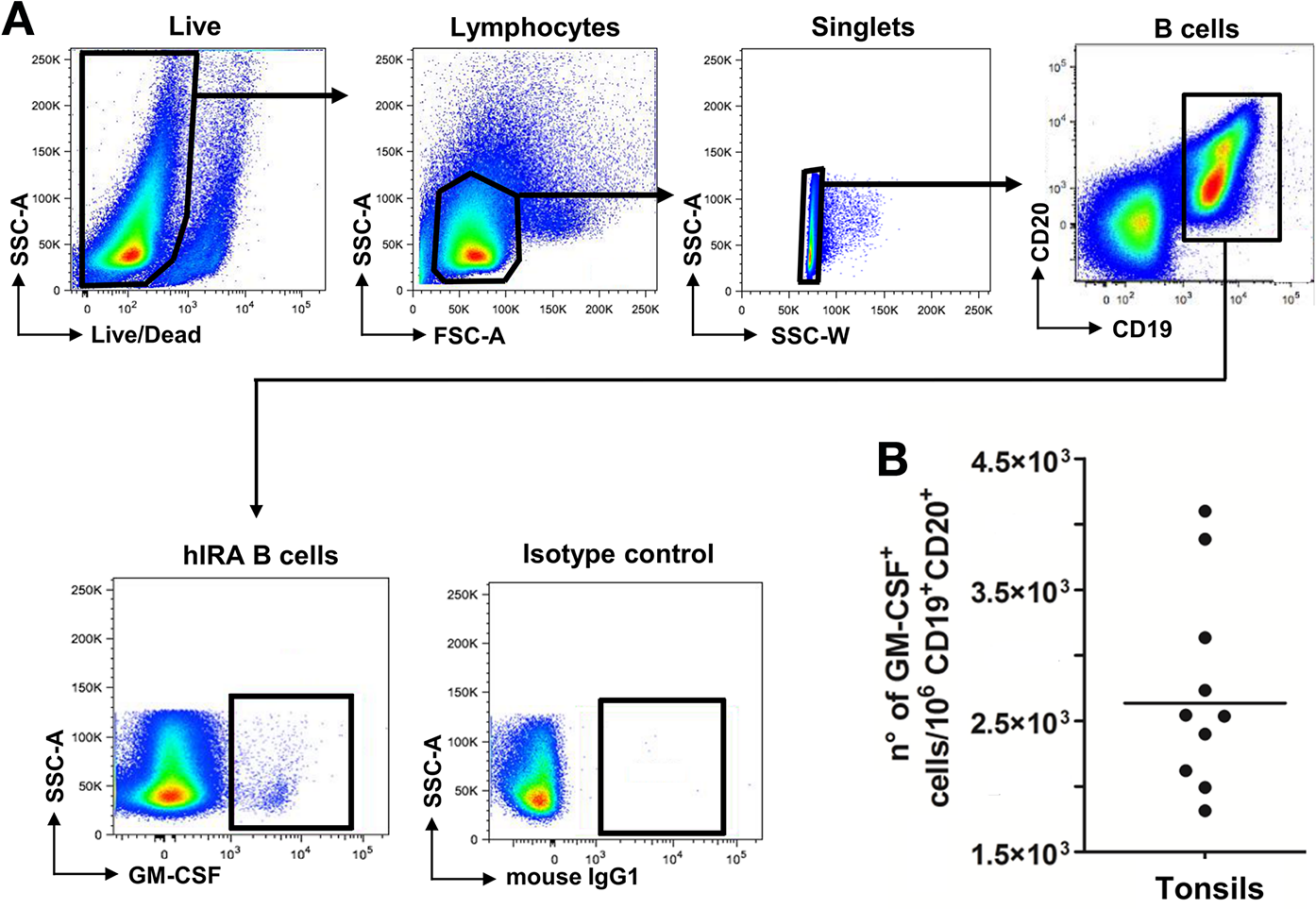
hIRA B cells are present in human tonsils. A) Gating strategy for the identification of hIRA B cells in tonsils. Tonsil-derived cells were obtained from tonsils of individuals undergoing routine tonsillectomy. After processing in the presence of DNAse and collagenase, samples were stained, fixed and analyzed by flow cytometry. Live cells were discriminated using Aqua Live/Dead staining kit, and singlet lymphocytes were selected on the basis of forward (FSC) and side (SSC) scatters. B cells were further identified as CD19^+^CD20^+^ and analyzed for the expression of GM-CSF. B) Quantification of hIRA B cells in tonsils. The number of CD19^+^CD20^+^GM-CSF^+^ cells was normalized on the number of total CD19^+^CD20^+^ lymphocytes after isotype background subtraction. The bar represents the geometric mean.

**Fig 2:**
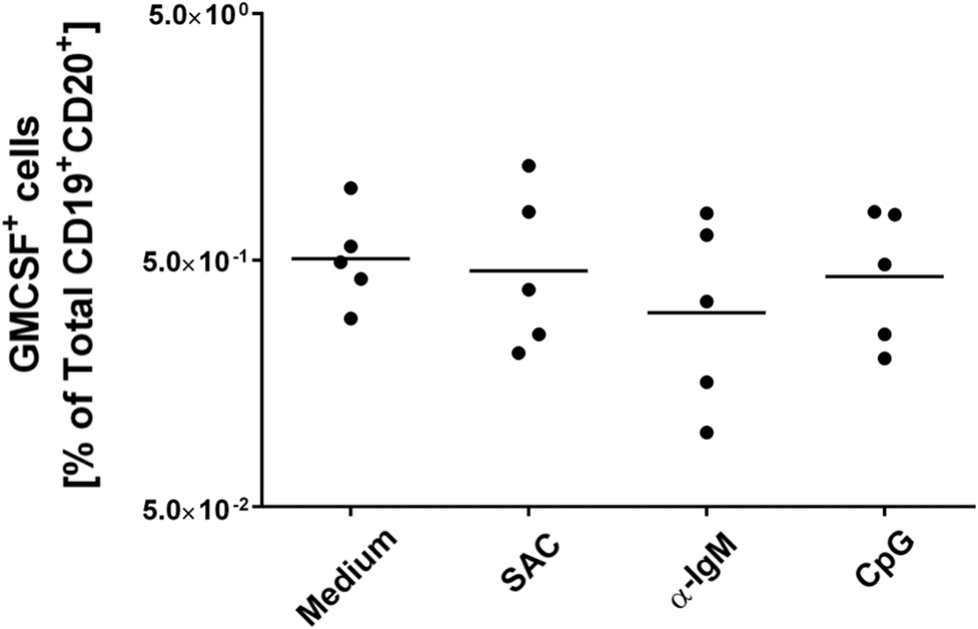
Effect of different stimuli on the frequencies of CD19^+^CD20^+^GM-CSF^+^ cells. *Ex-vivo* cells from tonsils (5 x 10^5^ cells/well) were stimulated for 2 days at 37°C in the presence of IL-2 with or without polyclonal stimuli (anti-IgM 10 μg/ml or SAC 1:20.000 final) or TLR9 ligand (CpG at 10 μg/ml). Brefeldin A was added 3 h before fixation and staining.

**Fig 3:**
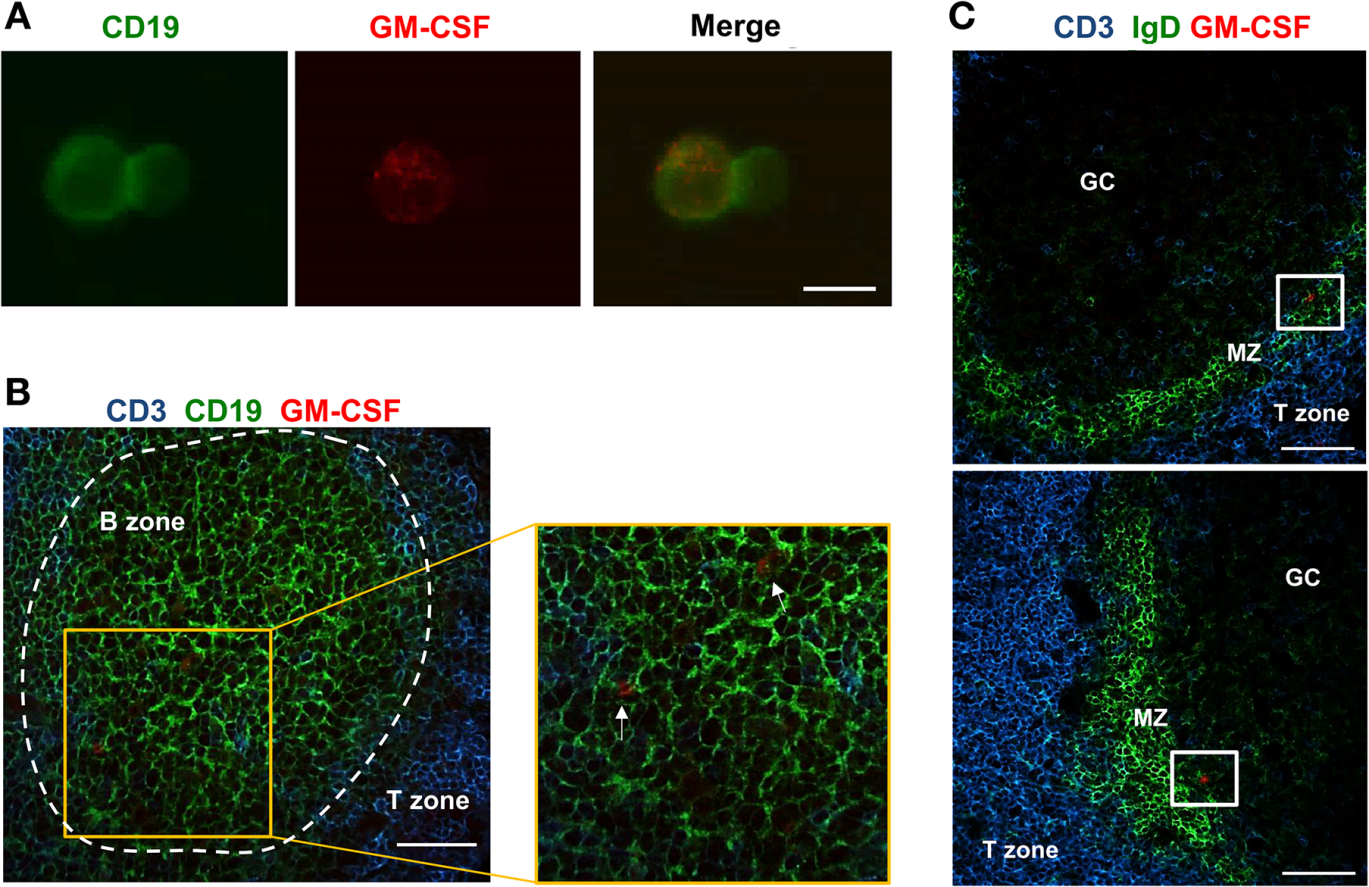
Human IRA B cells reside within the follicles. A) Cell suspensions from tonsils were seeded onto poly-L-lysine coverslips and fixed with formaldehyde. Cells were incubated for 1 h with anti-CD19 (Green) and anti-GM-CSF (Red). The merge panel shows the co-localization of GM-CSF and CD19. The picture is representative of three different subjects. Scale bar = 10 μm. B) 8-μm tonsil tissue sections were fixed with formaldehyde and stained using anti-CD3 (T cell area, Blue), anti-CD19 (B cell area, Green) and anti-GM-CSF (Red). The enlargement shows the presence of GM-CSF^+^ cells within B cell follicles (white arrows). The panel is representative of 3 independent experiments using different tonsils where many sequential sections were screened (n>5). Scale bar = 50 μm. C) 8-μm tonsil tissue sections were fixed with formaldehyde and stained using anti-CD3 (T cell area, Blue), anti-IgD (mantle zone, Green) and anti-GM-CSF (Red). The panel is representative of 3 independent experiments using different tonsils where many sequential sections were screened (n>5). Scale bar = 50 μm.

The recent identification of a population of innate-like B lymphocytes capable of producing GM-CSF, the so-called IRA B cells, in mouse peritoneal and pleural cavities, and in mouse and human spleens [5] [7] [8] led us to investigate the presence of hIRA B cells in tonsils, which represent one of the major secondary lymphoid organs in humans. While little information is available on their detection in the spleens of humans undergoing splenectomy [5], no data is available on the presence of IRA B cells in other lymphoid tissues. Therefore, we focused on tonsils from subjects undergoing tonsillectomy. Tonsil-derived cells were obtained as single-cell suspensions and analyzed by flow cytometry. To identify hIRA B cells, here defined as CD19^+^CD20^+^GM-CSF^+^ cells, the gating strategy showed in Fig 1A was adopted. Sizeable numbers of hIRA B cells were identified and are reported in Fig 1B. It is interesting to note that hIRA B cells were detected in 100% of the tonsils analyzed. Considering the low frequencies of hIRA B cells observed, we wanted to exclude a possible contamination by T cells, monocytes or plasma cells. As shown in S2 Fig, lower panel, hIRA B cells do not express CD3 (T cells) as well as CD14 (monocytes) or CD38 (plasma cells). GM-CSF production by hIRA B cells did not require a stimulation step *in vitro*, probably because of the chronically inflamed environment of the tonsils at the time of tonsillectomy. To confirm this hypothesis, we incubated tonsil-derived cells for 2 days with polyclonal stimuli (SAC, anti-IgM) or Toll-like receptor (TLR) agonist (CpG). Even under these conditions, we did not observe a significant increase in the frequencies of GM-CSF^+^ hIRA B cells (Fig 2). It is also important to note that the number of hIRA B cells in frozen, thawed and freshly prepared samples did not change (S1 Fig). Using confocal microscopy, CD19^+^GM-CSF^+^ cells were clearly visible in single-cell suspensions from tonsils; GM-CSF was well distributed within the cytoplasm (Fig 3A). To better characterize the distribution of GM-CSF^+^ hIRA B cells within the tonsil, tissue cryosections were stained with fluorescent anti-GM-CSF, anti-CD3 and anti-CD19 antibodies to discriminate T and B cell zones, respectively. As shown in Fig 3B, CD19^+^GM-CSF^+^ cells were localized in the B cell follicles. The analysis of several serial sections from different tonsils (n>4) never localized hIRA B cells out of the follicles, suggesting that the localization of these cells is restricted to the B cell areas. In addition, hIRA B cells were detected predominantly within the mantle zone of B cell follicles (IgD^+^ area) and not in GCs (IgD^-^ area) (Fig 3C).

### Phenotypic analysis of hIRA B cells

**Fig 4:**
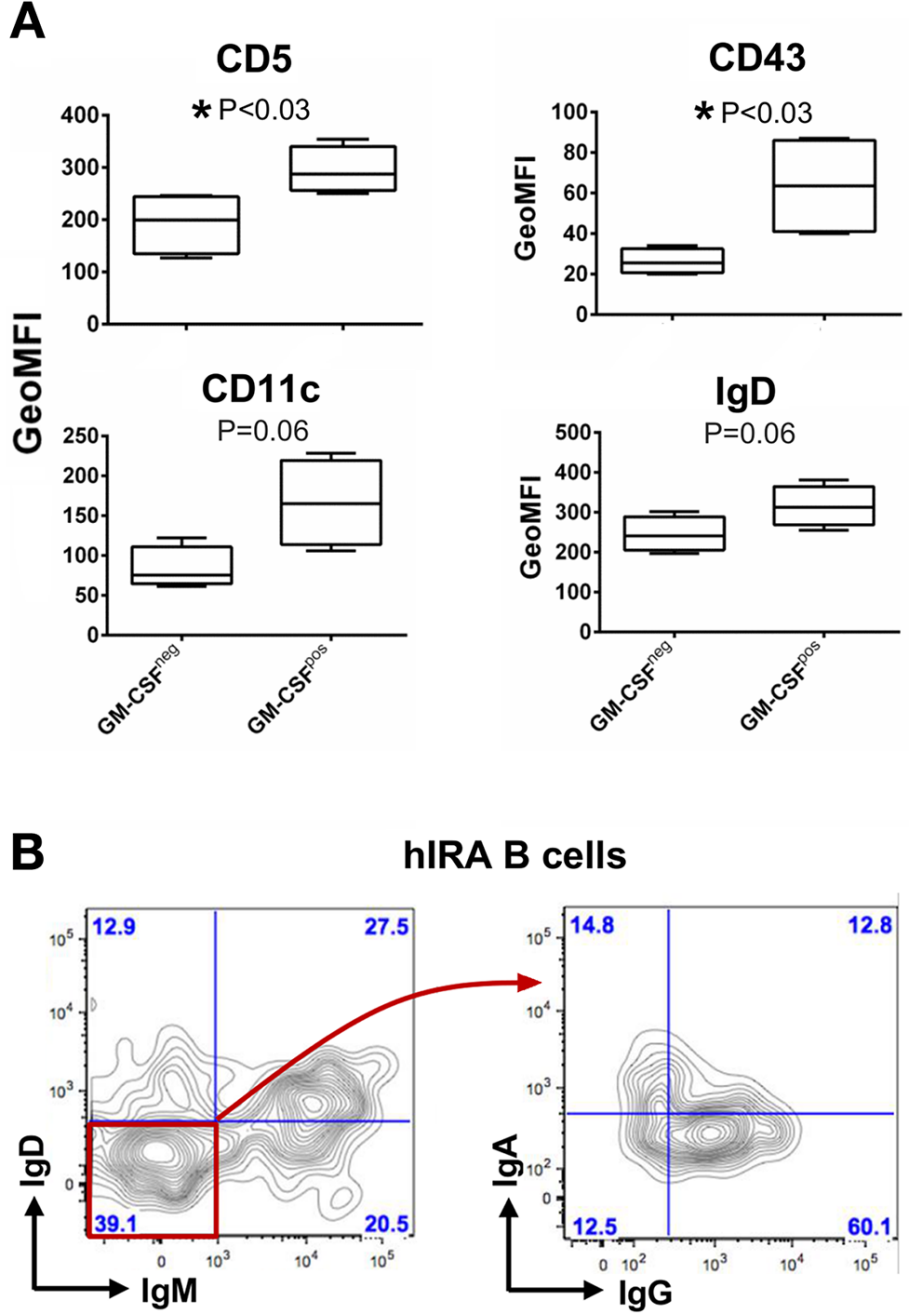
Phenotypic properties of hIRA B cells from tonsils. Conventional CD19^+^CD20^+^GM-CSF^-^(GM-CSF^neg^) and hIRA CD19^+^CD20^+^GM-CSF^+^(GM-CSF^pos^) B cells in cell suspensions from tonsils were analyzed by flow cytometry. A) Expression of CD5, CD43, CD11c and IgD in GM-CSF^neg^ versus GM-CSF^pos^ cells. The graphs show the geometric mean of fluorescence intensity (GeoMFI) for each marker after isotype background subtraction. Means and standard deviations are shown. P value was calculated using the non-parametric Mann–Whitney test (n = 4). B) Ig subclasses were analyzed within the hIRA B cell population. The plots are representative of n = 4.

The phenotypic properties of tonsil hIRA B cells were analyzed by flow cytometry evaluating the expression of a panel of surface markers modified by Rauch *et al*. [5] (Table 1). Since the ability to produce GM-CSF is considered the key feature of IRA B cells both in mice and in humans, we used this cytokine to discriminate hIRA B cells (defined as CD19^+^CD20^+^GM-CSF^+^) from conventional B lymphocytes (defined as CD19^+^CD20^+^GM-CSF^-^). Typical mature B cell markers CD19 and CD20 were clearly expressed by hIRA B cells; expression of CD40 and the chemokine receptor CXCR4 was also detected in all the samples analyzed (Table 1). We observed that levels of expression of CD5 and CD43 on hIRA B cells higher than those of conventional B cells were statistically significant, and detected a clear tendency for higher expression of IgD on hIRA B cells (Fig 4A). Conversely, no significant differences were observed in the expression of CD27, IgM, CD284 (TLR4) and CD86 when comparing hIRA B cells and conventional B cells (S3 Fig). Although lymphoid-specific glycoprotein CD5 is constitutively expressed in all T cells, this marker clearly identifies B1-a B cells in mice [9]. It is still unclear whether CD5 can be considered a specific marker of B-1 cells in humans, also because the existence of a human counterpart of the murine B-1 B cells is still a matter of debate [2,10–13]. It is known that CD5^+^ B cells are present in healthy individuals [14], and human CD5^+^ B cells can produce polyreactive auto-antibodies, accounting for 75% of B cells in the umbilical cord blood [15,16]. The data presented here support the evidence that, although CD5 cannot be considered an exclusive marker of human B-1 B cells, a higher expression level appears to be specific of hIRA B cells. CD43 is a surface molecule important for cell-matrix interactions, is expressed in humans by T cells, monocytes, and some B cells [17][18][19], and is considered a B-1 B cell marker both in mice and humans [2,10,11]. Compared to conventional B cells, the slightly higher expression of CD43 reinforces the hypothesis that hIRA B cells could be related to human B-1 B cells subset. The expression of integrin CD11c, which is typical of myeloid cells (DCs and monocytes), was also higher in hIRA B cells, although the difference was not statistically significant (Fig 4A). Furthermore, the analysis of Ig classes (Fig 4B) showed that the majority of hIRA B cells are IgM^+^, IgD^+^, or IgM^+^IgD^+^, indicating that hIRA B cells are predominantly non-switched B cells. Considering the current classification of B cell sub-populations in tonsils as transitional B cells (CD38^+^IgD^+^), naïve B cells (CD38^dim^IgD^+^CD27^-^), germinal center B cells (CD38^+^IgD^-^), memory B cells (CD38^dim^CD27^+^IgG^+^) and plasma cells (CD38^hi^CD27^+^CD43^+^), data reported in our work suggest that the majority of hIRA B cells seem to be part of the small fraction of CD19^+^CD38^-^IgD^+^CD27^-^ B cells and appear to be excluded from GC-dependent B cell maturation processes [20]. Our data on phenotypic characterization has shown that hIRA B cells are a heterogeneous population; therefore hIRA B cells have been defined by their capacity to produce measurable levels of GM-CSF. This parallels with the current understanding of B-10 B cells which produce IL-10 but do not express unique phenotypic or lineage markers useful for their identification [21]. However, the expression of CD5, CD43, CD20, together with the capacity to produce GM-CSF and the non-switched Ig profile (IgM^+^IgD^+^), allow us to conclude that the cell population characterized for the first time in human tonsils very likely represents the human counterpart of murine IRA B cells.

**Table 1:** Phenotypic properties of mouse and human IRA B cells.

Markers	Mouse (Rauch et al. 2012)	Human (present study)
	Frequency of positive cells	
CD5	++	++
CD11b	-	-
CD11c	n.a.	++
CD19	+++	+++
CD20	+++	+++
CD27	n.a.	+
CD40	n.a.	++
CD43	+++	+
CD86	n.a.	+
CD93	+	+
CD183 (CXCR3)	n.a.	-
CD184 (CXCR4)	n.a.	++
CD185 (CXCR5)	n.a.	-
IgM	+++	++ *
IgD	+	++ *
IgA	-	+
IgG	-	+
GM-CSF	+++	+++

Phenotypic properties of IRA B cells (CD19^+^CD20^+^GM-CSF^+^). In the present study, all markers are reported for cells taken ex vivo and unstimulated. Different tonsil samples were analyzed (n = 9).

+ = Low frequency (≤10%);

++ = Moderate frequency (≤50%);

+++ = High frequency (≥50%);

- = no expression.

n.a. = not assessed.

The asterisk (*) indicates variable frequencies between donors.

### hIRA B cells internalize *S*. *aureus in vitro*

**Fig 5:**
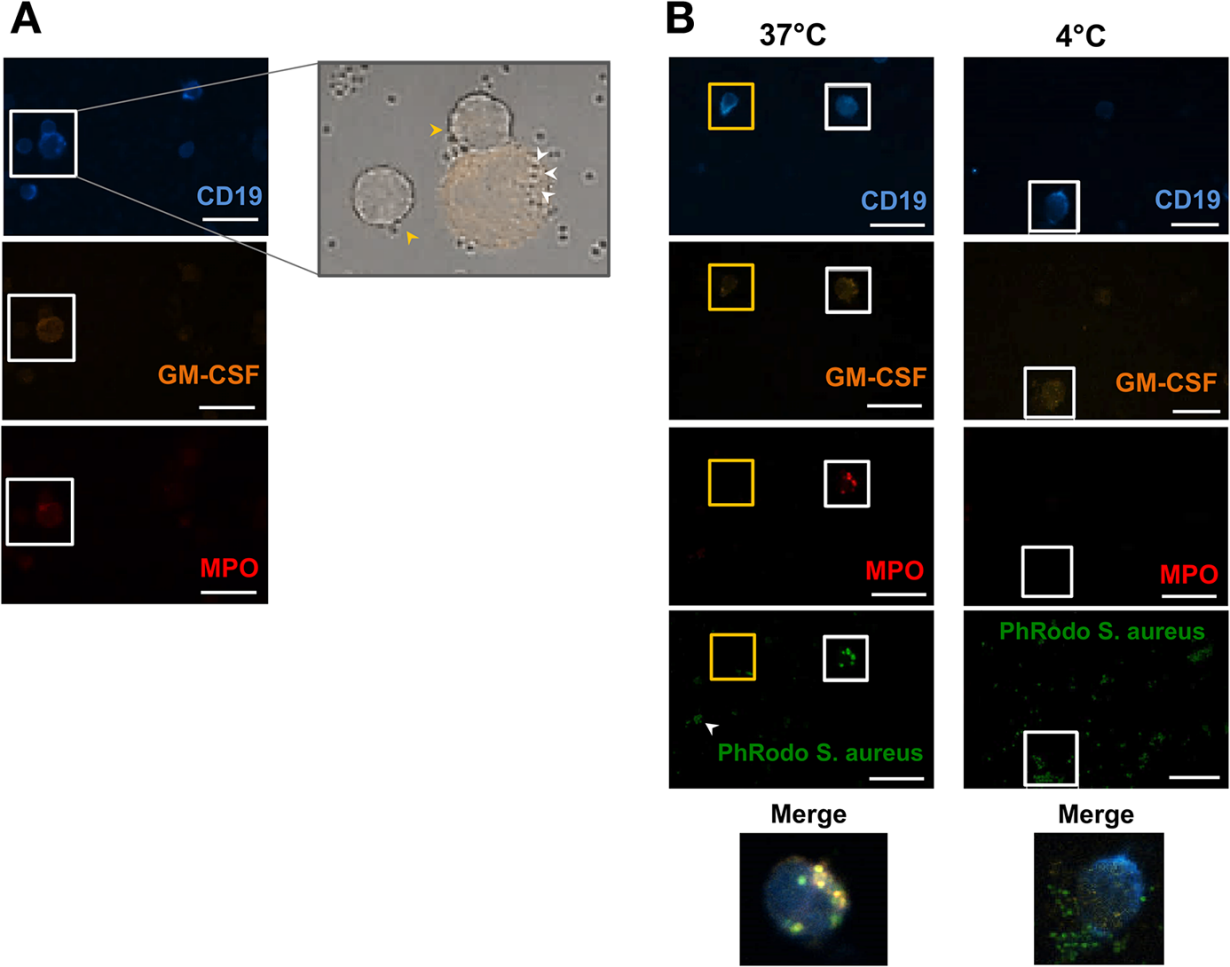
hIRA B cells have phagocytic capacity. A) Tonsil-derived cells were incubated for 2 h at 37°C with heat-inactivated *S*. *aureus* at 1:10 (cells:bacteria) MOI. After staining with anti-CD19 (Blue), anti-GM-CSF (Orange), and anti-MPO (Red), different optical fields were analyzed by confocal microscopy. The enlargement (bright field merged with the GM-CSF channel, right panel) shows the presence of *S*. *aureus* internalized by a CD19^+^GM-CSF^+^MPO^+^ cell (white arrows) and two CD19^+^GM-CSF^-^MPO^-^ cells with non-internalized bacteria (yellow arrows). B) *Left panels*: similar experimental approach described for panel A) with the addition of PhRodo labelled *S*. *aureus* bioparticles (green). The white arrows indicate not-internalized bioparticles (low fluorescence intensity), while bright fluorescent bioparticles localize within a CD19^+^GM-CSF^+^MPO^+^ cell (white square) but not within a conventional CD19^+^ cell (yellow square); *right panels*: control experiment where cells were incubated with PhRodo labelled *S*.*aureus* bioparticles (dark green) for 2 h at 4°C. Data are representative of 3 independent experiments. Scale bar = 10 μm.

Murine IRA B cells are considered a transitional B-1a-derived subpopulation of B lymphocytes possibly involved in reducing intestinal infections (LPS sepsis models) [5,6], and pneumonia [8]. However, the function of IRA B cells in humans is not known. B-1 cells have been recently reported to exert phagocytic capacity using mouse [22][23] and human B cell lines [24]. Since palatine tonsils are often colonized by bacterial pathogens such as *S*. *pyogenes*, *S*. *aureus*, and *H*. *influenzae*, we hypothesized that hIRA B cells might display such a phagocytic role. To address this question, tonsil-derived cells were co-cultured with heat-inactivated *S*. *aureus* (strain ST22), and confocal microscopy analyses were performed after staining with anti-CD19, anti-GM-CSF, and anti-myeloperoxidase (MPO) (Fig 5A). Interestingly, more than 80% of total CD19^+^GM-CSF^+^ cells co-expressed the lysosomal enzyme MPO, suggesting that hIRA resemble phagocytic cells in the composition of cytoplasmic granules. Indeed, hIRA MPO^+^ B cells were able to internalize *S*. *aureus* bacteria. To further substantiate this observation, tonsil-derived cells were incubated with PhRodo labelled *S*. *aureus* bioparticles, which dramatically increase the emitted fluorescence under the acidic conditions of the lyosome compartment. As showed in Fig 4B (left panels), brightly green fluorescent *S*. *aureus* bioparticles were found only inside the CD19^+^GM-CSF^+^MPO^+^ B cell population. Non-internalized *S*. *aureus* bioparticles were observed with a lower fluorescence green signal than the internalized ones. No CD19^+^GM-CSF^-^ cells were found to contain intracellular bacteria. On the contrary, no *S*. *aureus* bioparticles were found within hIRA B cells when cell:bacteria co-incubations were performed at 4°C (Fig 5B right panels).

Previous studies in mice reported that MPO is expressed by B-1a peritoneal B lymphocytes [23]. In the present study, we showed that *S*. *aureus* bacteria are actively internalized by GM-CSF^+^CD19^+^ hIRA B cells within an acidic MPO^+^ compartment, and that this activity was exclusively restricted to hIRA B cells. The uptake of bacteria by tonsil hIRA B cells required a metabolically active phagocytic process, as the uptake was not observed when the experiment was carried out at 4°C. These findings suggest that hIRA B cells may have innate effector functions by entrapping invading pathogens in acidic compartments.

In mice, antigens can be captured in the periphery by DCs, macrophages and neutrophils, and subsequently presented to MZ B cells secreting IgM by a T-independent pathway. In these conditions, secretion of GM-CSF by IRA B cells was suggested to enhance the survival and activation of antigen-presenting cells in the splenic red pulp [25]. GM-CSF is one of the most important growth factors acting on innate immune cells such as DCs, monocytes and macrophages. It has also been recently demonstrated that GM-CSF produced by innate-like cells activates both the formation of neutrophil extracellular traps and splenic marginal zone B cells in mice [26]. Whether or not these functions may apply to human hIRA B cells remains to be elucidated. In addition, considering that GM-CSF is currently used as vaccine adjuvant in Phase I and II clinical studies to overcome poorly immunogenic antigens such as those associated with intracellular infections and cancer [27], it would be interesting to address the role of IRA B cells in vaccination.

In conclusion, the data presented in this work show for the first time that hIRA B cells (*i*) are present in human tonsils; (*ii*) exclusively reside within the follicular areas; and (*iii*) can actively phagocyte bacterial microorganisms. Further work is required to clarify a possible relationship between hIRA and follicular B cells, the role of hIRA in the adaptive immune response, and the potential cellular targets of GM-CSF within the tonsils. Additionally, somatic hypermutation (SHM) analysis of Ig genes would be required to clearly define the nature (naïve or memory) of hIRA B cells.

## Supporting Information

S1 Fig Comparison between hIRA B cells detected in fresh samples and frozen/thawed samples. Numbers represent CD19^+^CD20^+^GM-CSF^+^ cells normalized on total CD19^+^CD20^+^ lymphocytes after isotype background subtraction. The bars represent the geometric means. No statistical difference was observed between fresh and frozen samples.
(TIF)

S2 Fig Gating strategy to investigate the possible presence of non-B cells within the hIRA B cell population. Expression of CD3 (T cells), CD14 (monocytes) and CD38 (plasmacells) was analyzed in hIRA B cells. Flow cytometry plots showed that hIRA B cells are single positive for CD19.
(TIF)

S3 Fig Comparison of surface molecules expression between hIRA and conventional B cells. Flow cytometry plots of CD27, IgM, IgD, CD284 (TLR4),CD86, CD43, CD5 and CD11c were shown as percentage (panel A) or as GeoMFI (panel B) on total CD19^+^GMCSF^+^ cells or CD19^+^GMCSF^-^ cells. In panel B, we reported only the graph for CD27 and IgM, since the expression of CD86,CD284 (TLR4) was very low. Means and standard deviations are shown. P value was calculated using the non-parametric Mann–Whitney test (n = 4).
(TIF)
