# Supplementary figures and images for: Innate Response Activator (IRA) B Cells Reside in Human Tonsils and Internalize Bacteria In Vitro
Source: PLoS One. 2015 Jun 12;10(6):e0129879. doi: 10.1371/journal.pone.0129879 (PMC4466315; doi:10.1371/journal.pone.0129879)

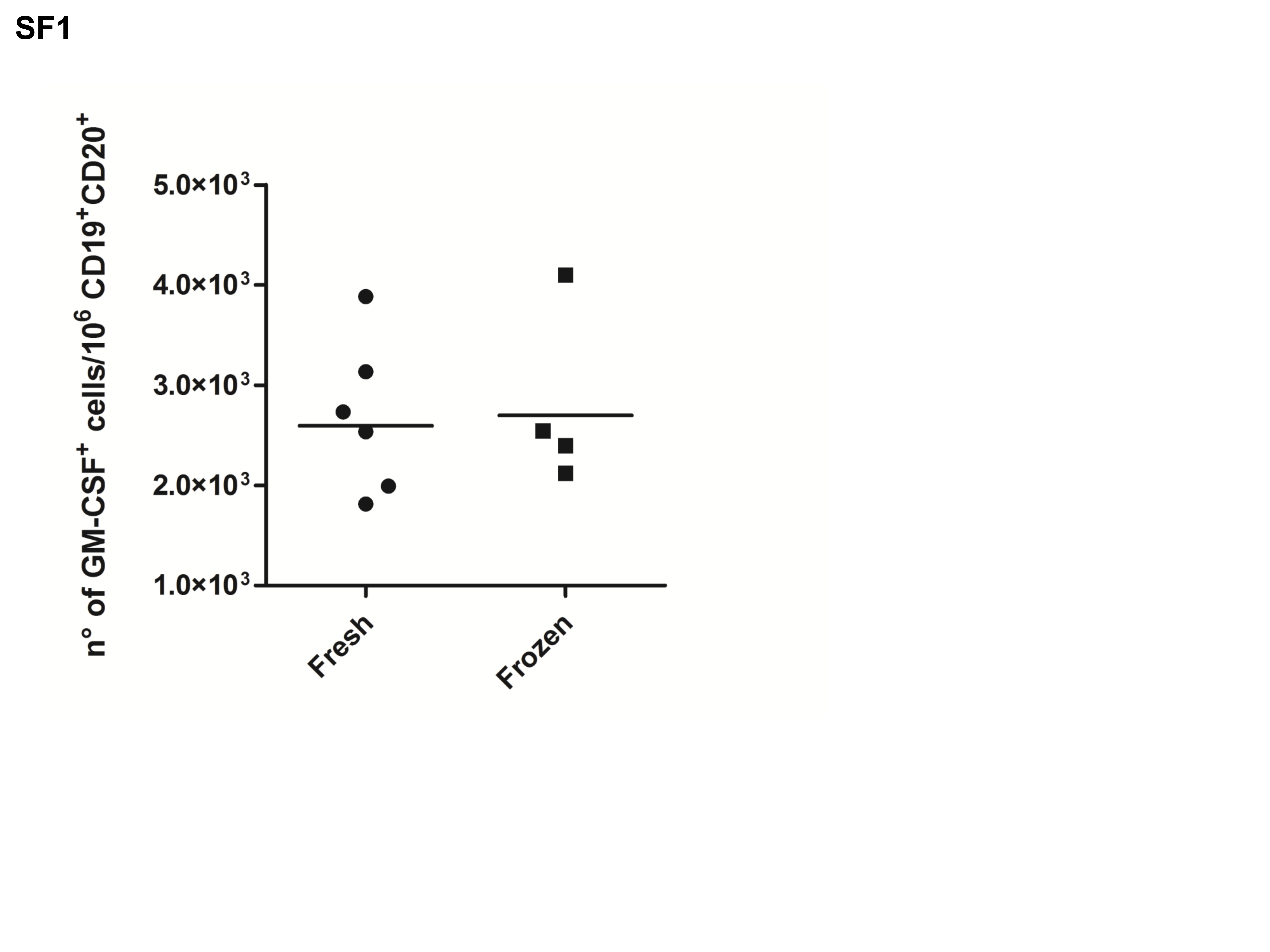

Supplement: S1 Fig — Numbers represent CD19+CD20+GM-CSF+ cells normalized on total CD19+CD20+ lymphocytes after isotype background subtraction. The bars represent the geometric means. No statistical difference was observed between fresh and frozen samples. (TIF) [file pone.0129879.s001.tif]

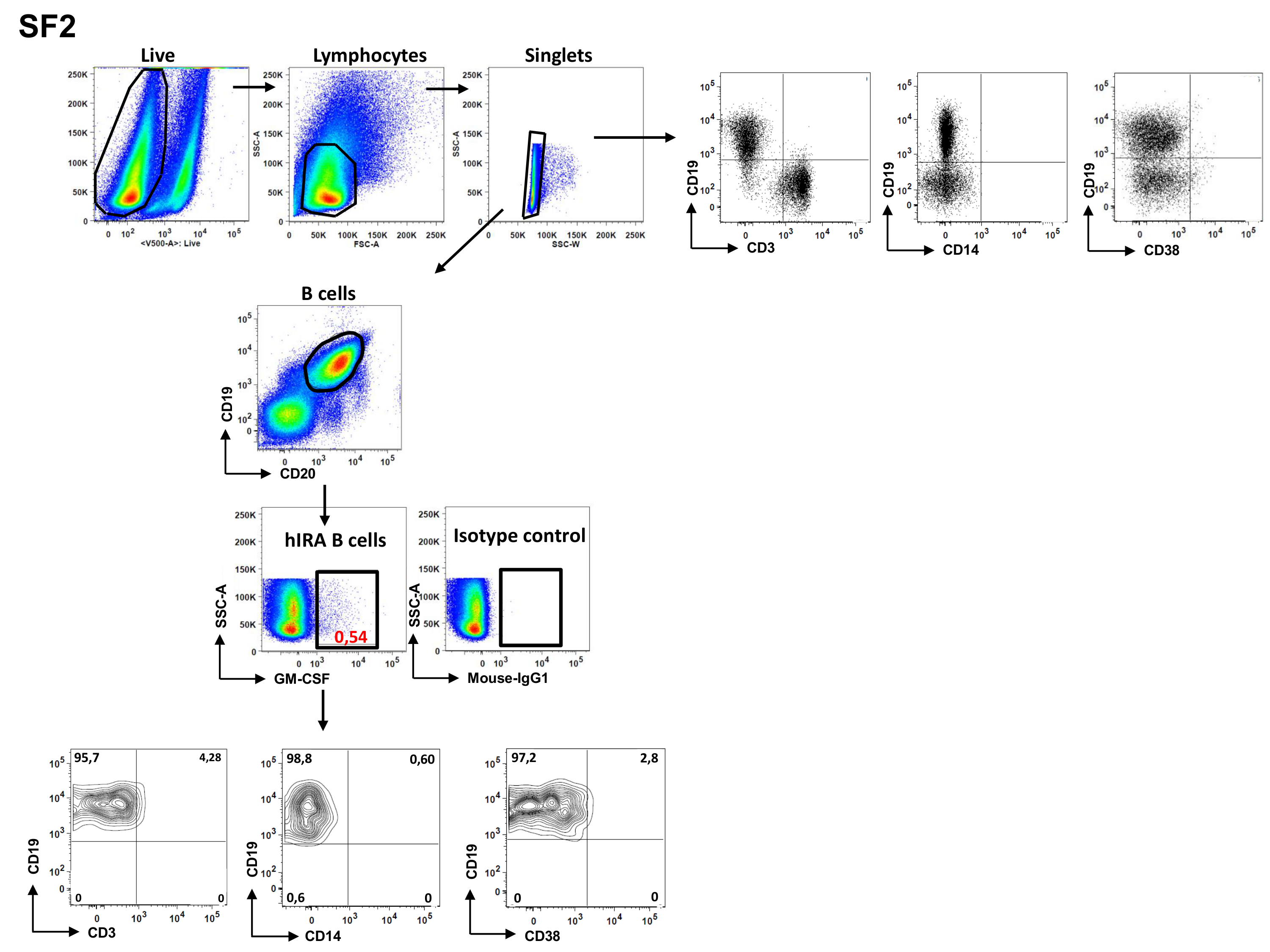

Supplement: S2 Fig — Expression of CD3 (T cells), CD14 (monocytes) and CD38 (plasmacells) was analyzed in hIRA B cells. Flow cytometry plots showed that hIRA B cells are single positive for CD19. (TIF) [file pone.0129879.s002.tif]

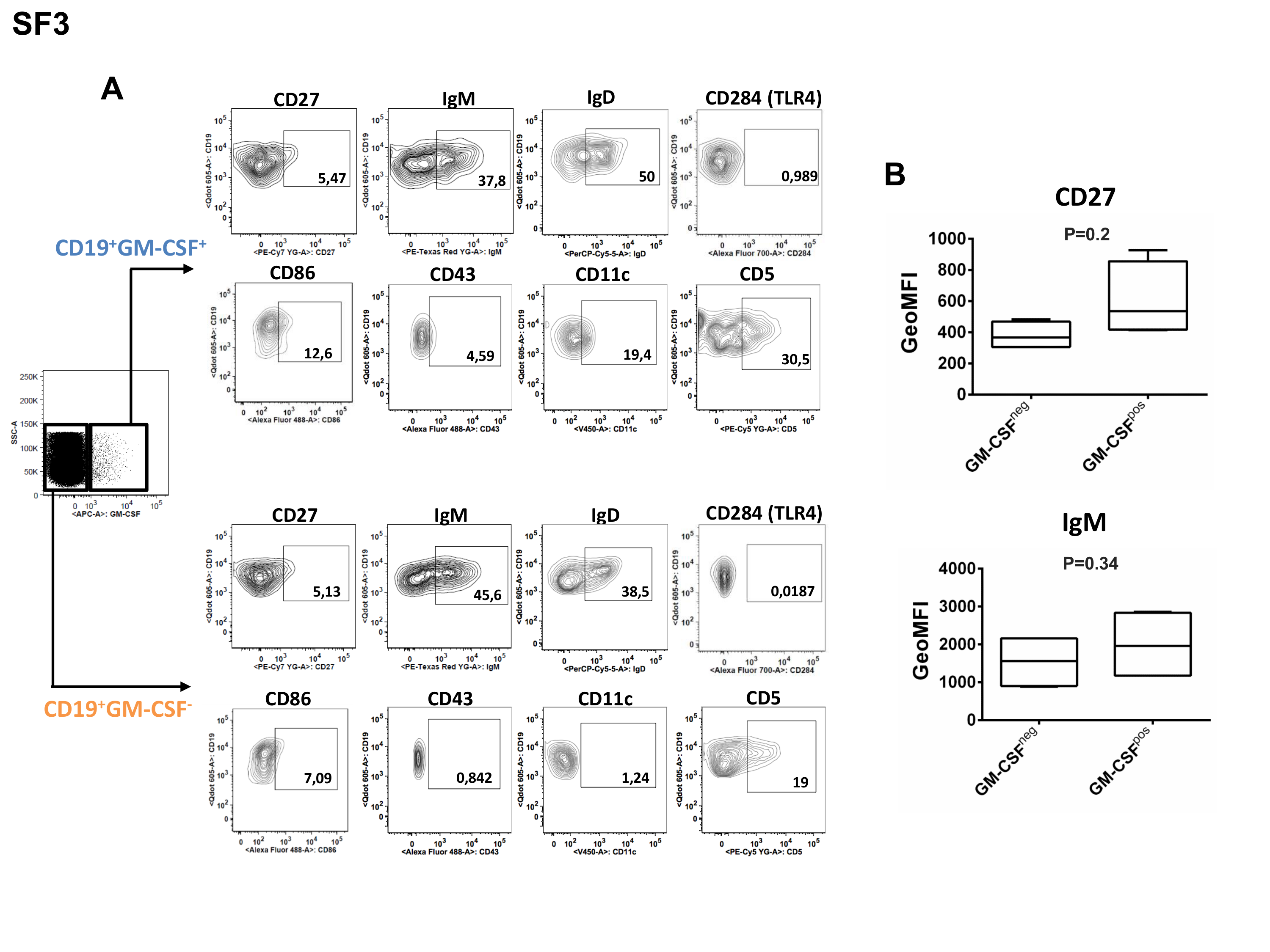

Supplement: S3 Fig — Flow cytometry plots of CD27, IgM, IgD, CD284 (TLR4),CD86, CD43, CD5 and CD11c were shown as percentage (panel A) or as GeoMFI (panel B) on total CD19+GMCSF+ cells or CD19+GMCSF- cells. In panel B, we reported only the graph for CD27 and IgM, since the expression of CD86,CD284 (TLR4) was very low. Means and standard deviations are shown. P value was calculated using the non-parametric Mann–Whitney test (n = 4). (TIF) [file pone.0129879.s003.tif]
